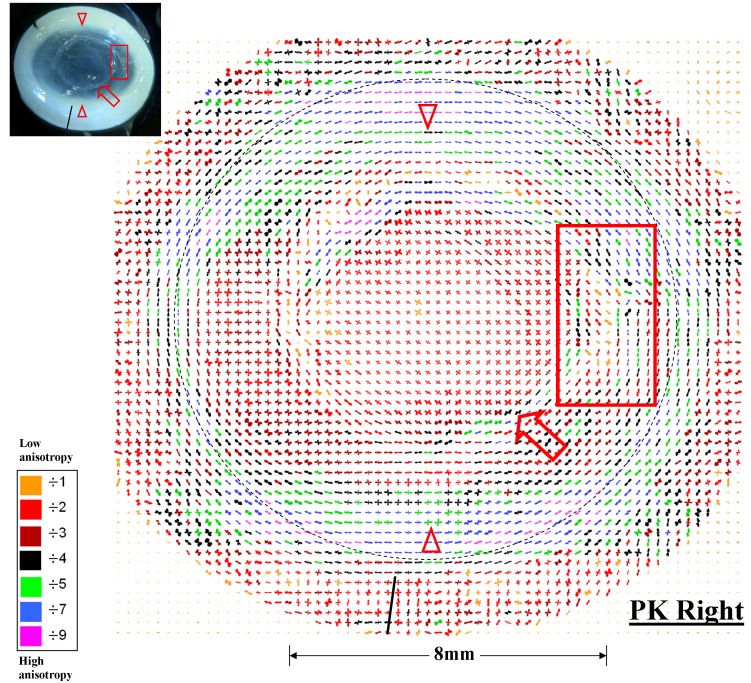# Correction: Quantification of Collagen Ultrastructure after Penetrating Keratoplasty – Implications for Corneal Biomechanics

**DOI:** 10.1371/annotation/1ca782a6-c5c1-4ef8-9c08-ca8ae0cdbaa6

**Published:** 2013-09-24

**Authors:** Craig Boote, Erin P. Dooley, Steven J. Gardner, Christina S. Kamma-Lorger, Sally Hayes, Kim Nielsen, Jesper Hjortdal, Thomas Sorensen, Nicholas J. Terrill, Keith M. Meek

The images for Figure 4, 5, and 6 were placed in the wrong locations. The legends for these figures are correct, and the correct figures are available below:

Figure 4: 

**Figure:**
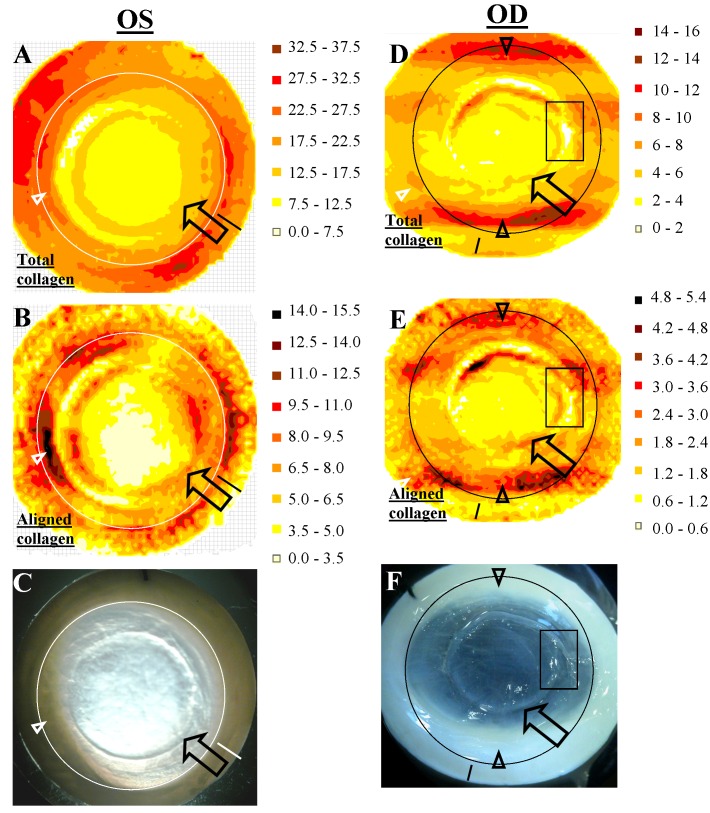


Figure 5: 

**Figure:**
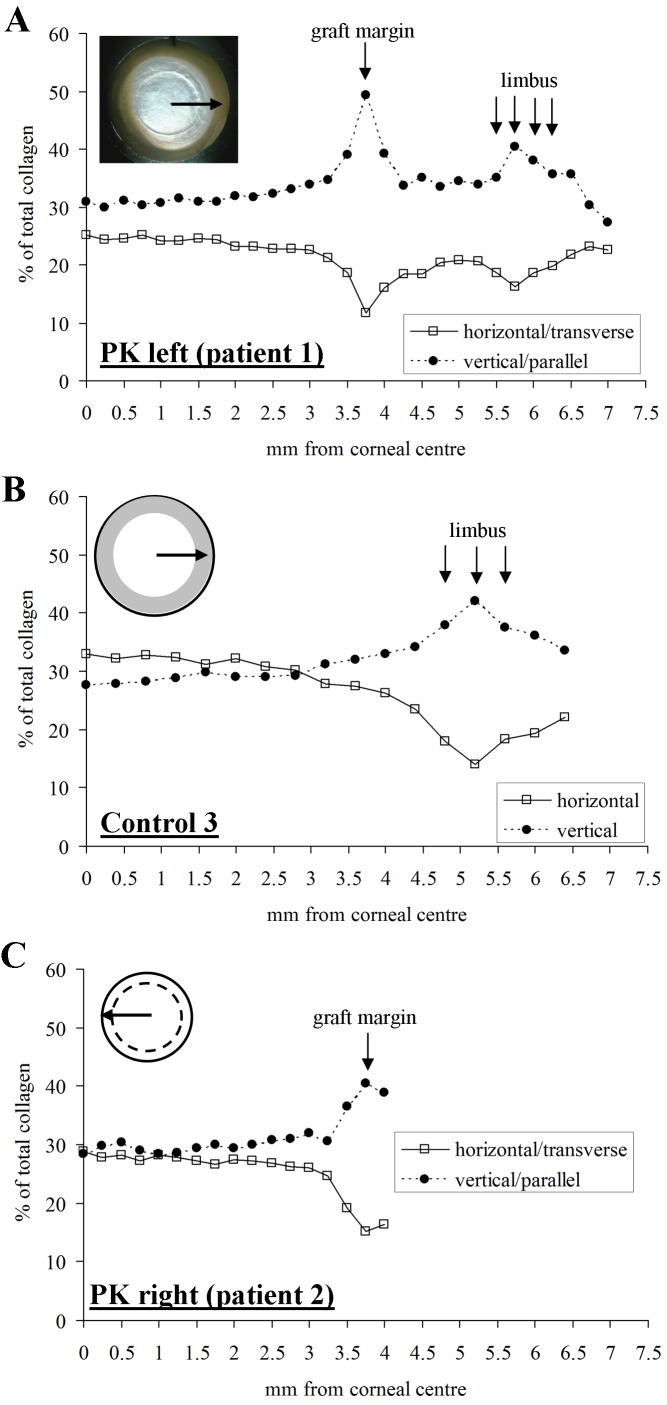


Figure 6: 

**Figure:**