# Correction: Quantification of Collagen Ultrastructure after Penetrating Keratoplasty – Implications for Corneal Biomechanics

**DOI:** 10.1371/journal.pone.0093246

**Published:** 2014-03-18

**Authors:** The PLOS ONE Staff

## Notice of Republication

This article was republished on February 28, 2014, to replace an incorrectly published version. The new version includes the figures in the correct order, addressing all issues noted in the previous Formal Correction. The originally published, uncorrected article and the republished, corrected article are provided here for reference.

## Supporting Information

File S1 Originally published, uncorrected article

File S2 Republished, corrected article
