# Supplementary material for: Correction: Quantification of Collagen Ultrastructure after Penetrating Keratoplasty – Implications for Corneal Biomechanics
Source: PLoS One. 2014 Mar 18;9(3):e93246. doi: 10.1371/journal.pone.0093246 (PMC3958550; doi:10.1371/journal.pone.0093246)
Supplement: File S2 — Republished, corrected article [file pone.0093246.s002.pdf]

PLOS | ONE

Quantification of Collagen Ultrastructure after Penetrating Keratoplasty – Implications for Corneal Biomechanics

Craig Boote[1]¶, Erin P. Dooley[1]¶, Steven J. Gardner[1], Christina S. Kamma-Lorger[1], Sally Hayes[1], Kim Nielsen[2], Jesper Hjortdal[2], Thomas Sorensen[3], Nicholas J. Terrill[3], Keith M. Meek[1]*

1 Structural Biophysics Group, School of Optometry and Vision Sciences, Cardiff University, Cardiff, United Kingdom, **2** Department of Ophthalmology, Aarhus University Hospital, Aarhus, Denmark, **3** Diamond Light Source, Didcot, Oxfordshire, United Kingdom

Abstract

Purpose: To quantify long-term changes in stromal collagen ultrastructure following penetrating keratoplasty (PK), and evaluate their possible implications for corneal biomechanics.

Methods: A pair of 16 mm post-mortem corneo-scleral buttons was obtained from a patient receiving bilateral penetrating keratoplasty 12 (left)/28 (right) years previously. Small-angle x-ray scattering quantified collagen fibril spacing, diameter and spatial order at 0.5 mm or 0.25 mm intervals along linear scans across the graft margin. Corresponding control data was collected from two corneo-scleral buttons with no history of refractive surgery. Wide-angle x-ray scattering quantified collagen fibril orientation at 0.25 mm (horizontal)×0.25 mm (vertical) intervals across both PK specimens. Quantification of orientation changes in the graft margin were verified by equivalent analysis of data from a 13 year post-operative right PK specimen obtained from a second patient in a previous study, and comparison made with new and published data from normal corneas.

Results: Marked changes to normal fibril alignment, in favour of tangentially oriented collagen, were observed around the entire graft margin in all PK specimens. The total number of meridional fibrils in the wound margin was observed to decrease by up to 40%, with the number of tangentially oriented fibrils increasing by up to 46%. As a result, in some locations the number of fibrils aligned parallel to the wound outnumbered those spanning it by up to five times. Localised increases in fibril spacing and diameter, with an accompanying reduction in matrix order, were also evident.

Conclusions: Abnormal collagen fibril size and spatial order within the PK graft margin are indicative of incomplete stromal wound remodelling and the long term persistence of fibrotic scar tissue. Lasting changes in collagen fibril orientation in and around PK wounds may alter corneal biomechanics and compromise the integrity of the graft-host interface in the long term.

Citation: Boote C, Dooley EP, Gardner SJ, Kamma-Lorger CS, Hayes S, et al. (2013) Quantification of Collagen Ultrastructure after Penetrating Keratoplasty – Implications for Corneal Biomechanics. PLoS ONE 8(7): e68166. doi:10.1371/journal.pone.0068166

Editor: Michael E. Boulton, University of Florida, United States of America

Received April 16, 2013; **Accepted** May 30, 2013; **Published** July 5, 2013

Copyright: © 2013 Boote et al. This is an open-access article distributed under the terms of the Creative Commons Attribution License, which permits unrestricted use, distribution, and reproduction in any medium, provided the original author and source are credited.

Funding: Medical Research Council (Grants G0600755 and MR/K000837/1). The funders had no role in study design, data collection and analysis, decision to publish, or preparation of the manuscript.

Competing Interests: The authors have declared that no competing interests exist.

* E-mail: meekkm@cf.ac.uk

¶ These authors contributed equally to this work.

Introduction

The biomechanical properties of the cornea are influenced significantly by the organization of collagen fibrils that form the bulk of the corneal stroma [1]. Alterations in the alignment [2,3,4], diameter [5,6,7] and spatial order [5,6] of stromal fibrils occurs after penetrating injury, and there is evidence that some of these changes persist long-term in and around the wound margin following some types of corneal surgery [3,8,9]. Some of these findings may be related to the observation that the mechanical strength of corneal scar tissue never fully reaches uninjured levels [10]. A number of investigators have examined the appearance of the graft margin following penetrating keratoplasty (PK) [3,9,11,12,13,14,15]. All of these studies reported significant demarcation or abnormalities in the stroma at the graft-host interface, suggesting a limited stromal healing response. However, the majority of this literature featured either histological or confocal microscopic findings, while quantitative, ultrastructural studies of collagen architecture following penetrating corneal wounds remain scarce.

Here we have used x-ray scattering to quantify stromal collagen ultrastructure in a pair of post-mortem human eyes from a patient who underwent bilateral PK for keratoconus 12 (left eye) and 28 (right eye) years previously, and a 8.5 mm post-operative corneal button from the right eye of a second patient who required re-graft 13 years after originally undergoing PK. The availability of tissue

of this kind is extremely limited. This study therefore afforded us a rare opportunity to investigate the long-term effects of penetrating injury on corneal ultrastructure by carrying out the first quantitative study of collagen organisation across whole corneal specimens featuring PK wounds.

Methods

The research presented in this manuscript was approved by the Human Science Ethical Committee (School of Optometry and Vision Sciences, Cardiff University, UK) and the South East Wales Research Ethics Committee (Cardiff, UK). The institutional review board approved the use of all corneas described in this study; a waiver of consent was given for the donor corneas. All tissue used in this study was obtained in accordance with the tenets of the Declaration of Helsinki, and local ethical rules were adhered to throughout. All experimental procedures were performed in accordance with the Declaration of Helsinki.

PK Patient 1 Tissue Details

A pair of post-mortem eyes with history of bilateral PK for keratoconus was obtained from The Danish Cornea Bank, Aarhus University Hospital (Aarhus, Denmark). The age of the PK recipient at death was 79 yrs. The donors' ages were not available. 16 mm diameter corneo-scleral buttons were excised and the orientation within the eye marked with a scleral suture. The specimens were then immediately wrapped in polyvinylidene chloride film to prevent dehydration, frozen and stored at −80°C until the time of x-ray experiments. Inspection of the PK tissue using light microscopy prior to the x-ray experiments disclosed that both specimens displayed prominent regions of opacity in the peripheral cornea, located on the temporal side in the left eye and inferior/superior in the right, and that the graft margins were still visibly demarcated (Fig. 1A,C). Additionally, a marked discontinuity of the wound edge was visible on the temporal side of the right eye (Fig. 1C). *Left eye history:* Underwent 8.2 mm/8.0 mm PK for keratoconus 12 years previously. The apex was thinned and located inferior/temporal. However, thinning and astigmatism could not be accurately quantified due to irregularity of the corneal surface. 5 months after PK there was vascularization into the donor cornea as far as the sutures. The sutures (superior: 5 loops parallel to the suture, inferior: 4 loops) were removed after 12 months, and at 14 months there were no reported complications. *Right eye history:* Underwent PK for keratoconus 28 years previously. No further information was available.

PK Patient 2 Tissue Details

A 8.5 mm diameter post-operative corneal button from a 51 year old patient who required re-graft following suspected recurrence of keratoconus 13 years after initial 7.5 mm/7.25 mm PK surgery. A detailed structural examination of this specimen was published by our lab previously and concluded that corneal ectasia at 13 years may have recurred due to a mechanical failure of the graft rather than keratoconus recurrence [3]. In the current study, a portion of the raw x-ray scatter data was re-analysed in order to verify results from patient 1.

Control Tissue Details

Two post-mortem corneo-scleral buttons from donors aged 71 yrs (Control 1) and 47 yrs (Control 2), with no history of keratoconus or refractive surgery, were obtained from Bristol Eye Hospital (Bristol, UK). The specimens were wrapped in polyvinylidene chloride film, frozen and stored at −80°C until the time of x-ray experiments. Further control results were obtained via re-analysis of raw x-ray data from a normal cornea (Control 3) examined in detail in a previous publication by our lab [16].

Small-angle x-ray Scattering

Small-angle x-ray scattering [17] (SAXS) was performed on Beamline I22 at the Diamond Light Source (Didcot, UK), using an x-ray beam (wavelength: 0.1 nm) with a cross-sectional diameter of 0.25 mm. Each film-wrapped specimen was thawed and placed into sealed Perspex® (Lucite Group Ltd, Southampton, UK) chambers with Mylar® (DuPont-Teijin, Middlesbrough, UK) windows. The incident x-ray beam was directed towards the anterior specimen surface, perpendicular to the corneal apex, and the specimens were allowed to retain their natural curvature. Specimen alignment was achieved by an initial exposure of x-ray sensitive film placed in the specimen chamber to locate the position of the incident beam. SAXS patterns, each resulting from an x-ray exposure of 10 s, were collected along multiple linear scans across the graft-host interface of each specimen at, 0.25 mm (PK left) or 0.5 mm (PK right) sampling intervals (Fig. 1B,D), and recorded electronically on an x-ray detector placed 6 m behind the specimen position. Equivalent data from corresponding locations on the control specimens were also collected (Fig. 1E). Specimen translation between exposures was achieved using a motorized stage integrated with the x-ray camera shutter. Analysis of corneal SAXS patterns allows quantification of the average separation, diameter and index of spatial order of collagen fibrils in the stromal volume sampled by the x-ray beam [17]. Measurements of all three collagen parameters were obtained at each sampled point in the specimens, as described previously [18,19,20].

Wide-angle x-ray Scattering

Following SAXS experiments, the PK specimens were immediately placed in 4% paraformaldehyde and stored at 4°C for subsequent characterization using wide-angle x-ray scattering (WAXS). Our previous work has established that this mild fixation method does not affect corneal/scleral collagen parameters as measured by WAXS [16]. Diamond Beamline I02 was used to record WAXS patterns across the whole of each PK specimen at 0.25 mm (horizontal)×0.25 mm (vertical) intervals, using an x-ray beam of wavelength 0.098 nm and a cross-sectional diameter of 0.2 mm. For data collection each specimen was mounted in the same way as for SAXS, such that the incident x-ray beam was directed at the anterior surface and perpendicular to the corneal apex, and the tissue's natural curvature was retained. Initial specimen alignment was achieved via an in-line microscope directed along the incident x-ray beam direction. WAXS patterns, each resulting from an x-ray exposure of 8 s, were recorded electronically on a CCD detector (ADSC, Poway, USA) placed 550 mm behind the specimen position. Specimen translation was achieved using integrated motor stages.

Analysis of corneal/scleral WAXS patterns provides a quantitative measure of bulk collagen fibril orientation, as an average value within the stromal volume sampled by the x-ray beam [21]. For every sampled location in the PK specimens, we obtained three measurements: 1) the relative number of fibrils preferentially aligned at a given angle within the tissue plane (over and above the population of fibrils that are arranged isotropically), 2) the total x-ray scatter integral (a measure of the total mass of fibrous collagen), 3) the aligned x-ray scatter integral (a measure of the mass of preferentially aligned collagen). A detailed account of the data analysis may be found in a previous publication [21].

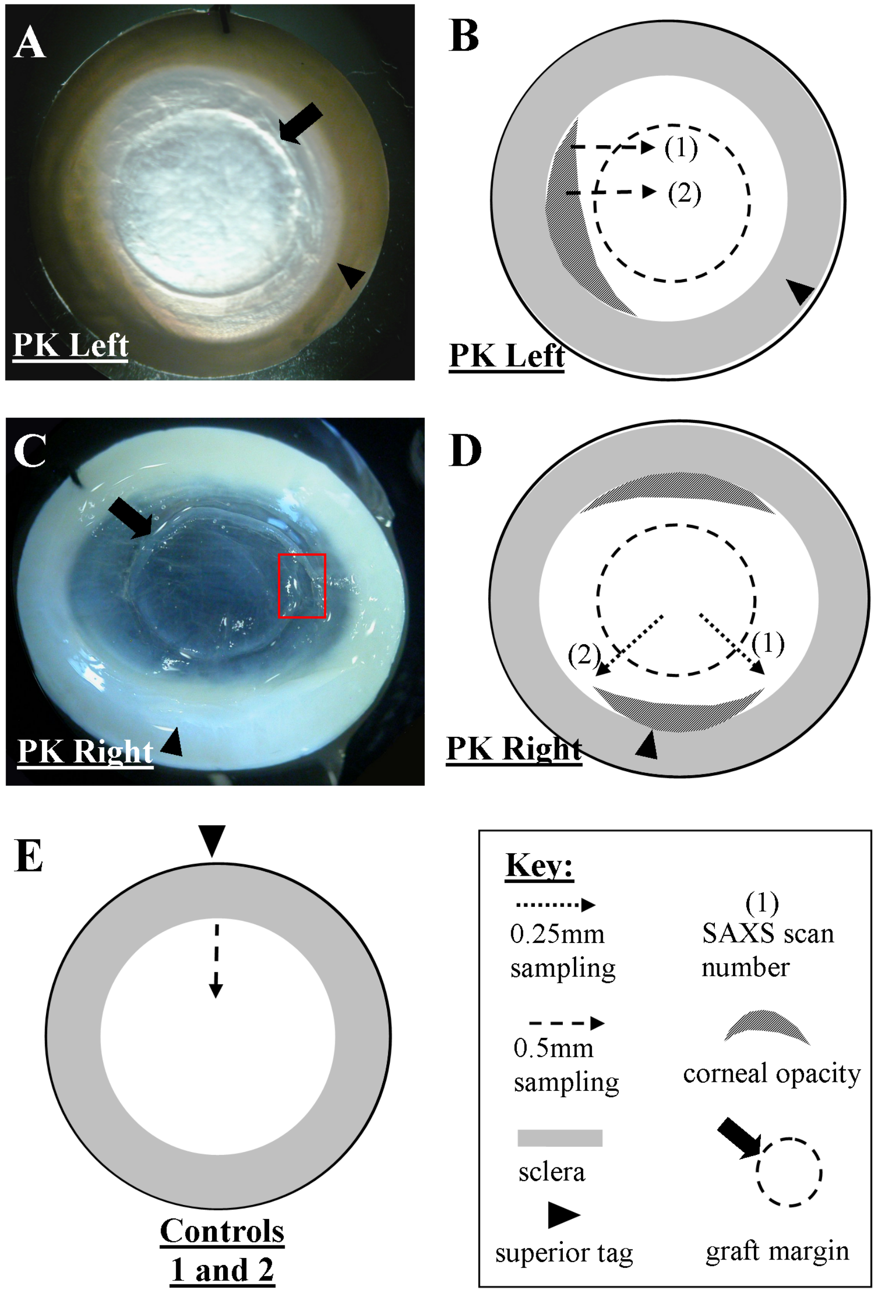

Figure 1. Appearance of specimens and SAXS sampling locations. A) Left and **C)** right PK specimens from Patient 1. Red rectangle in C) shows discontinuity of the wound edge in the right eye. **B–E)** SAXS scans performed on the PK and control specimens.
doi:10.1371/journal.pone.0068166.g001

Results

Collagen Spatial Organisation

Fig. 2 shows SAXS results from two linear scans across the graft-host interface of the left PK specimen of Patient 1. Similar to previous findings in normal human corneas using transmission electron microscopy [22] and SAXS [18,23], average fibril spacing and diameter generally displayed minimum values in the central cornea and increased with proximity to the limbus, with fibril order index displaying the opposite trend. However, notably, additional local elevations in fibril spacing and decreased levels of fibril spatial order were also consistently observed in the region of the graft margin. In some scans, these alterations were also accompanied by corresponding increases in fibril diameter. Fig. 2 also presents corresponding data from two scans across the graft margin of the right PK specimen from the same patient, in which similar observations were noted. Analysis of the control data confirmed that the localised alterations in fibril ultrastructure noted in the graft margin of both PK specimens were not present in corresponding regions of unwounded corneas (Fig. 2), consistent with previous studies of normal human tissue [18,22,23].

Collagen Orientation and Mass Distribution

Fig. 3 presents a map of predominant collagen fibril alignment across the left PK specimen, determined using WAXS. Each

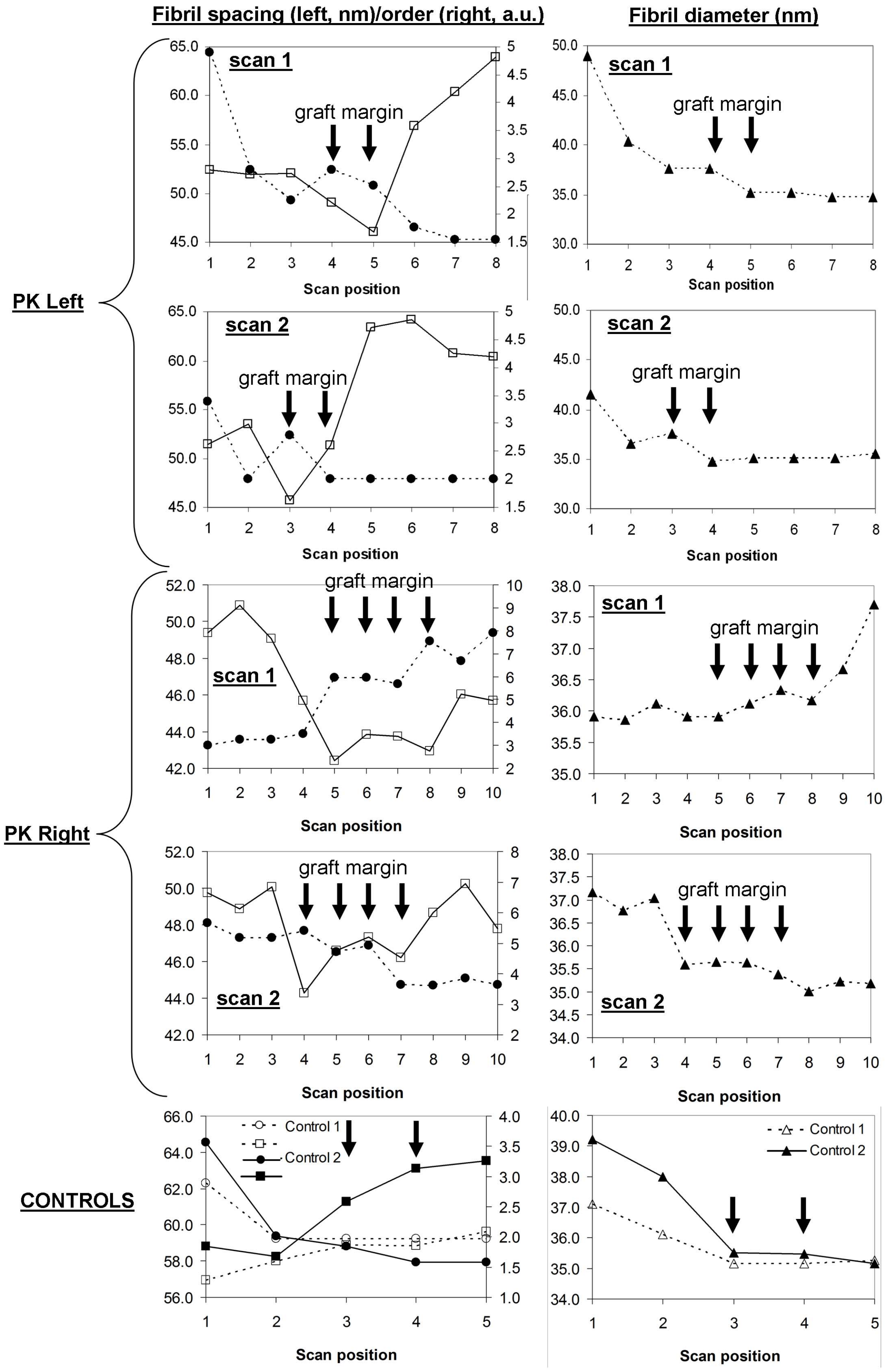
Fibril spacing (left, nm)/order (right, a.u.)
Fibril diameter (nm)
PK Left
PK Right
CONTROLS
scan 1
scan 2
graft margin
Scan position
Control 1
Control 2

Figure 2. SAXS data across the graft-host interface of the PK specimens from Patient 1, showing measurements of collagen fibril spacing (circles), spatial order index (squares) and diameter (triangles) along the scans indicated in Fig. 1. Corresponding data is also shown for control specimens. Arrows indicate scan positions lying on the PK graft margin and their equivalent positions on the controls. Note local increase in fibril spacing and diameter, and accompanying decrease in spatial order, in the graft margin for the PK specimens.
doi:10.1371/journal.pone.0068166.g002

sampled point is represented by an individual polar vector plot, in which the distance from the plot centre to periphery in a given direction represents the relative amount of preferentially aligned collagen lying in that direction [21]. The same technique has been used extensively to map collagen orientation across normal human corneas [16,24]. This previous work has shown that, centrally, the normal cornea is characterised by a preponderance of fibrils oriented along the superior-inferior and nasal-temporal meridians, while towards the periphery these preferred directions gradually alter to become predominantly tangential at the limbus [16,24]. Reference to Fig. 3 shows that this overall pattern was also evident in the left PK specimen. However several additional features, not present in normal tissue, were also noted.

Firstly, the circumferential collagen which characterises the normal human limbus and perilimbal sclera had extended into the peripheral cornea on the temporal side, corresponding in location

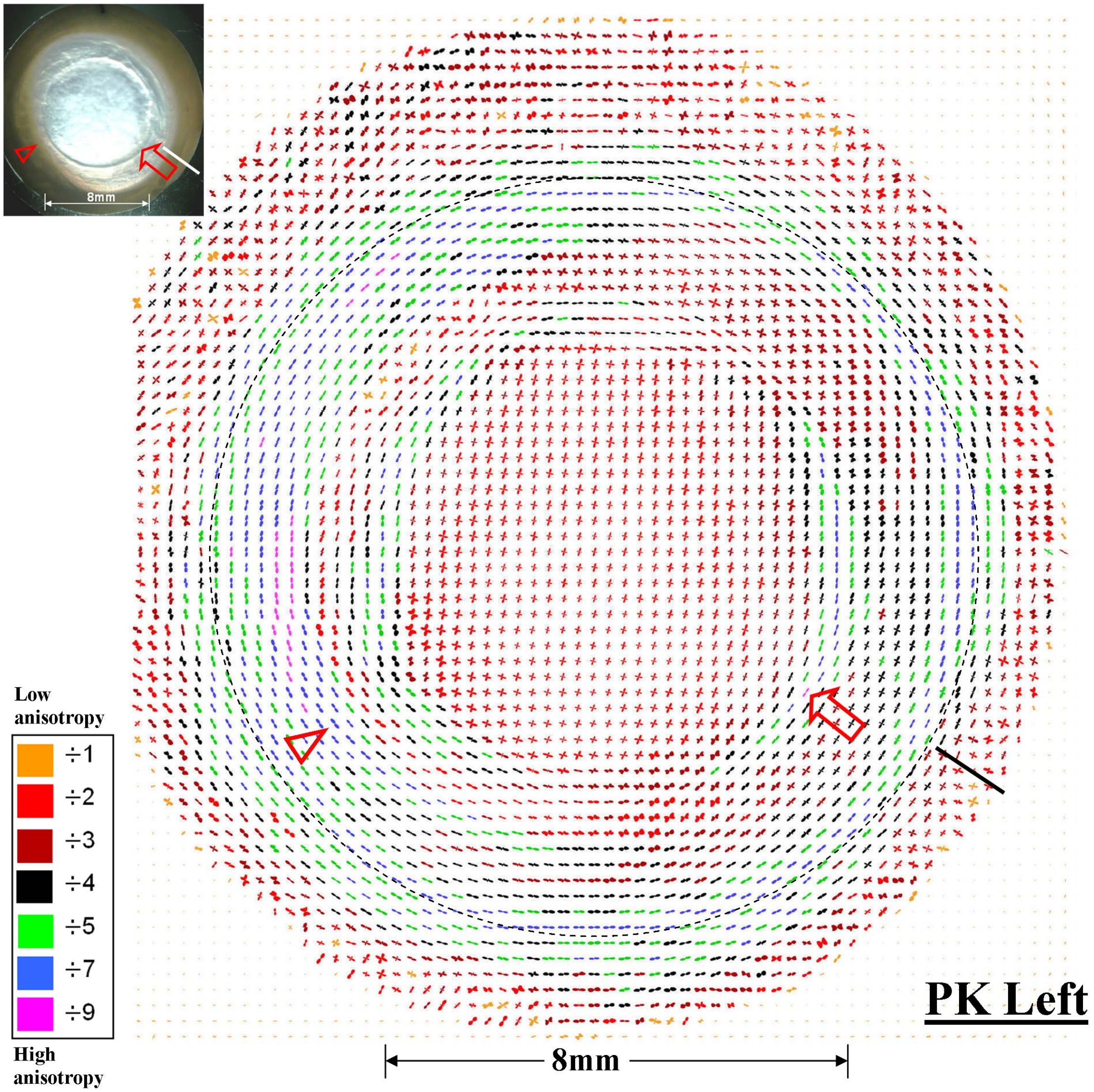

Figure 3. Polar vector map of preferential collagen fibril alignment across the left PK specimen of Patient 1, determined using WAXS. The larger plots (indicative of greater fibril alignment) have been scaled down for montage display by factors indicated in the colour key. Broken circle: limbus. Arrow: tangential fibril alignment along the graft margin. Arrowhead: abnormal inward extension of tangential limbal fibrils into the peripheral cornea, corresponding to region of prominent corneal opacity. Solid line: superior tag. Inset: location of noted features on actual specimen.
doi:10.1371/journal.pone.0068166.g003

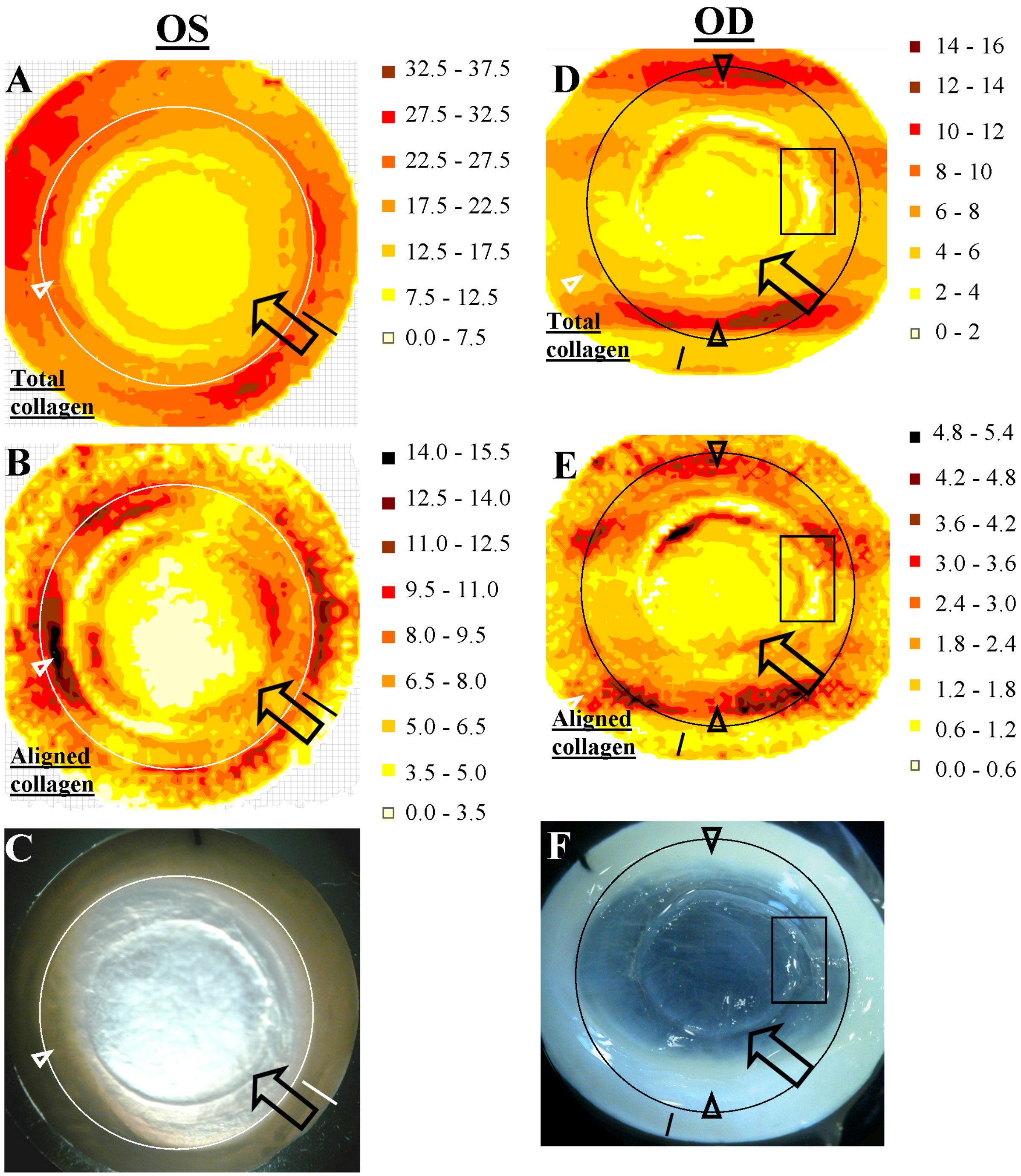

Figure 4. Distribution maps of A,D) total and B,E) preferentially aligned collagen across the PK specimens from Patient 1, determined using WAXS (arbitrary units). Arrows: local elevation in collagen mass at the graft margin. Arrowheads: abnormal elevation of collagen mass in the peripheral cornea, corresponding to the prominent regions of corneal opacity. Solid circles: limbus. Solid lines: superior tag. Rectangles: Elevated collagen mass delineates the separated wound edges of the right PK specimen, with a reduction in the intervening tissue. **C,F)** Location of noted features on actual specimens.
doi:10.1371/journal.pone.0068166.g004

with the region of prominent corneal opacity (Fig. 3 - arrowhead). This region was also notable for displaying elevated collagen mass in comparison with the nasal side of the eye (Fig. 4A–C - arrowheads), an increase which was proportionately greater for aligned, compared to total, collagen. Secondly, along the majority of the graft margin there were marked alterations to normal collagen orientation, characterised by fibrils aligned predominantly tangential to the wound edge (Fig. 3 - arrow). In this region an approximately two-fold local increase in total collagen scatter was measured (Fig. 4A - arrow), consistent with an overlap of the donor button and host bed. Aligned collagen scatter in this region showed a proportionately greater elevation, measuring up to four-fold higher than in adjacent regions inside and outside of the graft

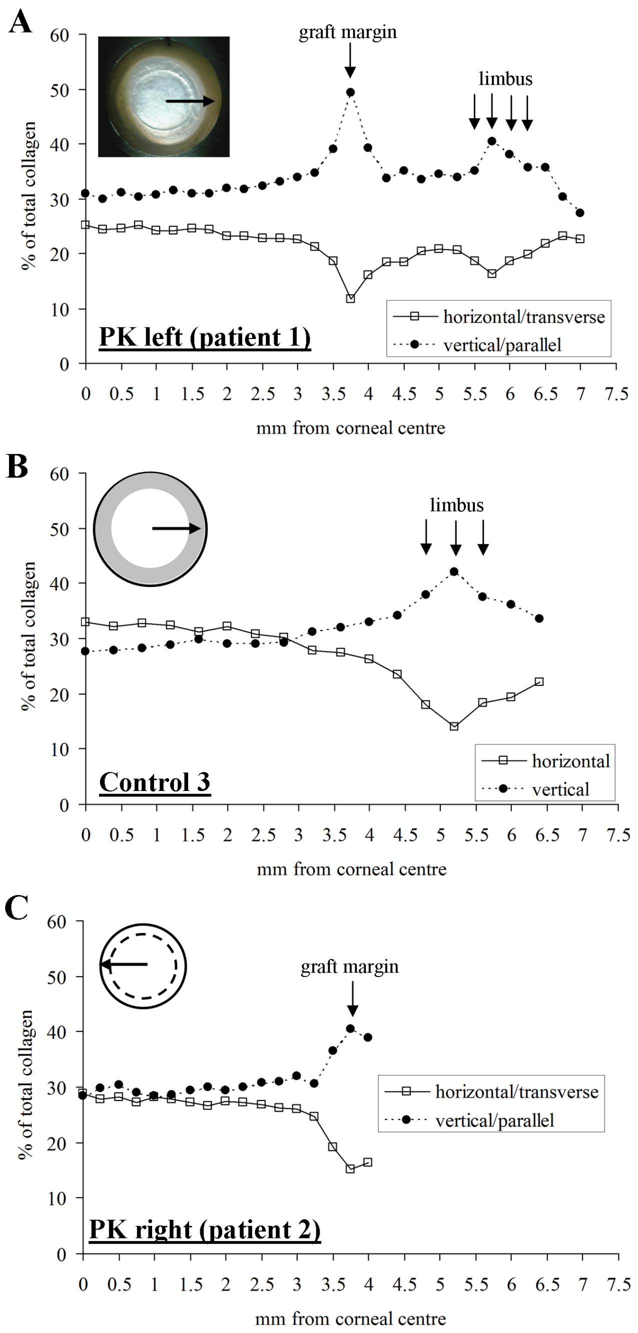

Figure 5. Proportion of total collagen oriented within 45° sectors of the horizontal (transverse to the wound edge) and vertical (parallel to the wound edge) directions as a function of distance from the corneal centre in A) Patient 1, left PK specimen (sampling interval: 0.25 mm), B) normal control cornea (sampling interval 0.4 mm) and C) Patient 2, right PK specimen (sampling interval: 0.25 mm). Note marked increase/decrease in collagen oriented parallel/transverse to the wound edges in A) and C).

doi:10.1371/journal.pone.0068166.g005

margin (Fig. 4B - arrow), indicating considerable reinforcement of aligned collagen around the wound edge.

In order to quantify further the change in normal alignment of collagen across the graft margin, we compared the relative proportions of collagen oriented transversely and parallel to the wound edge. This was done by calculating from the WAXS patterns [25] the proportion (relative to total collagen) of fibrils oriented within 45° sectors of the horizontal (i.e. transverse) and vertical (i.e. parallel) directions, when approaching the wound along a horizontal corneal semi-meridian (Fig. 5A - *inset*). These results are shown in Fig. 5A, and compared to equivalent data from a normal human cornea (Control 3) examined in a previous WAXS study [16] in Fig. 5B. The measurements disclose an abrupt 15% increase in the proportion of collagen aligned parallel to wound edge, and an accompanying 11% decrease in the proportion of transversely oriented collagen, at this particular location on the graft margin, resulting in approximately five times as much fibrous collagen edging the wound as that spanning it. Inspection of the corresponding raw x-ray scatter intensity values confirmed that this effect arose from a combination of a 46% absolute increase in parallel collagen and a 40% absolute decrease in transverse collagen (data not shown). A similar trend was confirmed at a corresponding location on the PK graft margin of Patient 2, in which the number of collagen fibrils edging the wound was approximately 3-fold higher than those spanning it (Fig. 5C).

Figs. 6 and 4D,E present maps of preferential collagen orientation and collagen mass distribution, respectively, across the right PK specimen. Within the donor button, two orthogonal dominant directions of collagen were again evident (Fig. 6). However, unlike the donor button of the left PK specimen (Fig. 3), and the central cornea of normal human eyes [16,24,26], these preferred directions did not correspond to the superior-inferior and nasal-temporal meridians of the recipient eye (Fig. 6), suggesting that in this case the donor button was grafted obliquely. The graft margin was again notable for the presence of markedly reinforced collagen aligned tangential to the wound edge (Figs. 6 and 4D–F - arrows). Furthermore, the visible discontinuity of the wound margin on the temporal side was clearly reflected in the pattern of collagen organisation in this region, with dominant fibril orientation and elevated collagen mass delineating the separated graft margin, and fibrillar distortion and reduction of collagen mass in the tissue between the separated wound edges (Figs. 6 and 4D–F - rectangles). The prominent regions of corneal opacity in the inferior and superior peripheral cornea were again characterised by an abnormal inward extension of the highly aligned circumferential limbal/perilimbal scleral collagen (Figs. 6 and 4D–F - arrowheads).

Discussion

Although PK is a highly successful long-term treatment option in cases of severe corneal scarring and advanced pathology, late-onset complications can present in a minority of patients. In the case of PK for advanced keratoconus, the indication presented by the patients studied herein, graft failure occurs in around 6% of cases at a mean time of 13 years post-op [27]. To what extent, if any, collagen structural changes contribute to the mechanisms underlying graft failure remains to be established. However, there is a large body of evidence to suggest that normal stromal architecture may never be fully recovered in full-thickness graft wounds [3,9,11,12,13,14,15,28,29]. The current results align well with this view, demonstrating extensive long-term abnormalities in collagen fibril orientation and spatial organisation around the entire graft margin following PK. Firstly, we noted that collagen orientation was predominantly tangential to the wound edge, consistent with findings reported in previous long-term PK follow-ups [3,9]. It is possible that this observation could be partly a legacy of a mechanical distortion of existing collagen during

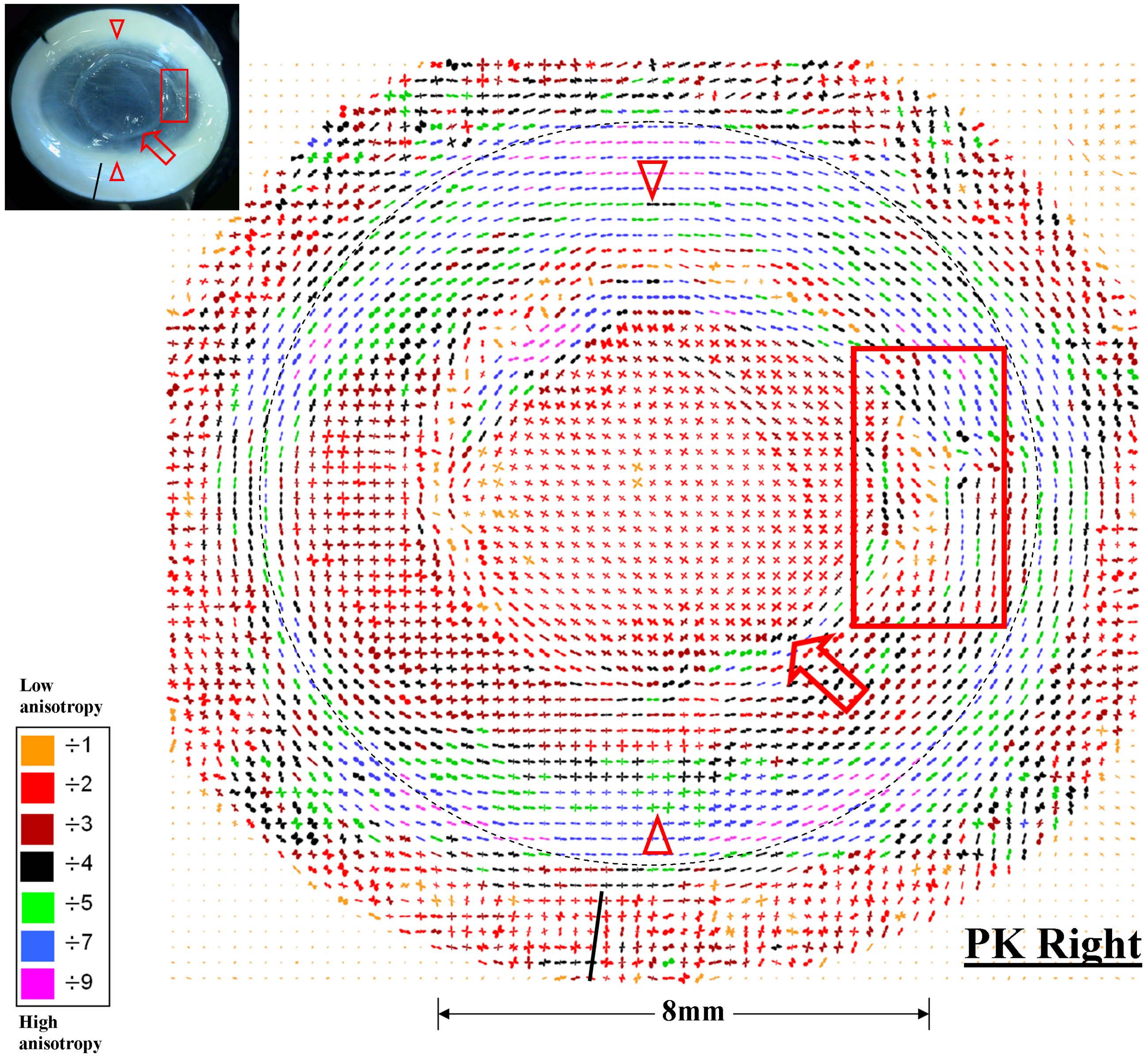

Figure 6. Polar vector map of preferential collagen fibril alignment across the right PK specimen of Patient 1, determined using WAXS. The larger plots (indicative of greater fibril alignment) have been scaled down for montage display by factors indicated in the colour key. Broken circle: limbus. Arrow: tangential fibril alignment along the graft margin. Arrowhead: abnormal inward extension of tangential limbal fibrils into the peripheral cornea, corresponding to regions of prominent corneal opacity. Rectangle: fibril alignment delineates separated wound edges and is disturbed in intervening tissue. Solid line: superior tag. Inset: location of noted features on actual specimen.
doi:10.1371/journal.pone.0068166.g006

trephination [2], likely augmented by new collagen secreted by infiltrating activated host keratocytes aligning with the donor button edge via contact guidance [30].

Significant changes in collagen alignment around the wound margin could impact on the mechanical stability of the graft-host interface. Specifically, at the examined location in the left PK graft margin of Patient 1, our measurements indicated that only around 10% of the total collagen was aligned transversely to the wound edge. Such a relative lack of meridional fibrils spanning the graft margin might be reasonably expected to encourage a wound under tension to reopen. In contrast, over 50% of the collagen at this point was aligned parallel to the wound edge. Notably, a predominantly circumferential arrangement of fibrils is predicted, from biomechanical considerations, to maximise out-of-plane corneal deformation under intraocular pressure [31]. This could potentially exacerbate the tendency of an already weakened donor-host tissue interface to separate. With this in mind, it is relevant to note that traumatic wound dehiscence has been reported up to 19 years after PK [11,29], while stress analysis experiments have indicated that the graft-host interface remains weak even after the tissue appears fully healed [28].

Although neither eye of Patient 1 examined herein suffered a reported failure, the wound margin of the patient's right eye did display a prominent visible separation on the temporal side, which was confirmed by the collagen orientation pattern in this region. It may be significant that this graft was performed earlier and hence had been in the eye some 16 years longer than the fellow eye, which showed no such changes. Moreover, we also noted a distortion of the collagen in the tissue between the separated wound edges, in which fibrils had aligned radially, i.e. along the direction of the apparent outward migration of the wound edge and perpendicular to the edge itself. The altered collagen alignment here may not have resulted in a significant local mechanical effect because the degree of anisotropy was generally

low (indicated by the orange color coding of the vector plots). However, interestingly a similar pattern of radial collagen alignment abutting the wound edge was previously noted by the same technique in the specimen from Patient 2, in which an initially successful PK graft had seemingly mechanically failed after 13 years [3]. With respect to these observations, it is unfortunate that no follow-up ophthalmic records were available for the right eye of Patient 1.

The long-term persistence of circumferential collagen in the graft margin we report here is compatible with the general view of stromal collagen after penetrating corneal injury having a limited capacity to remodel following initial wound repair, and this is further supported by our observation of altered collagen spatial organisation in the graft margin of Patient 1. Specifically, we noted localised increases in collagen spacing and diameter, and a reduced level of spatial order, observations which are all consistent with the presence of fibrotic scar tissue deposited early in wound healing [5,6,20]. This could be a contributing factor to the visible opacity of the wound margin in these specimens [32], and may also be expected to further alter the biomechanical properties of the tissue in this region [18].

A final observation of the current study of potential importance to corneal biomechanics is that, with reference to the dominant fibril directions of the donor and host collagen, the right PK donor button appeared to have been grafted obliquely. It has been suggested that the superior-inferior and nasal-temporal preferred collagen orientation in the central human cornea may reflect a mechanical adaptation of the tissue to forces imposed by the extraocular muscles [25,26,33,34]. In this context, it is interesting that the orthogonal fibril directions in the right PK specimen of Patient 1 examined herein had remained oblique to the rectus muscle insertions, with apparently no significant re-alignment after 28 years as may have been expected on the basis of the abovementioned criteria. This may reflect the extremely slow rates of collagen turnover in the quiescent stroma [35]. Alternatively, it could be linked to the observation that the inflation behaviour of the cornea under normal conditions has been shown to be insensitive to the collagen structure of the central region [36]. A further interpretation could be that any new collagen deposited during wound healing has been laid down mainly in register with the donor lamellae, possibly indicating that infiltrating cells have taken their directional cues largely from the existing collagen scaffold irrespective of mechanical stimuli.

The peripheral (keratoconic) tissue of both eyes of Patient 1 displayed prominent peripheral corneal opacity, located temporally in the left eye and inferior/superior in the right. The WAXS data from this these regions revealed abnormal levels of highly aligned tangential collagen, resembling that normally restricted to the limbus and perilimbal sclera [16,24]. Taken together with the physical appearance of the tissue, this suggests peripheral scleralization had occurred. To our knowledge there is no specific documented association of corneal scleralization and keratoconus. Therefore we contend that this may be a case of isolated peripheral sclerocornea which, as is the case here, usually presents bilaterally and asymmetrically [37], as opposed to an abnormality linked directly to the keratoconus itself.

In summary, this paper documents the first quantitative, long-term follow-up of collagen organisation across whole transplanted corneas. In principle, the changes noted in and around the graft margin could affect corneal biomechanical behaviour and graft stability. However further research, possibly focussing on the application of structural data in numerical simulation of corneal biomechanics, may help to establish any definitive link that may exist between the ultrastructural changes presented herein and the instances of PK graft dehiscence and mechanical failure documented in the clinical literature.

Acknowledgments

The authors thank Val Smith of the Bristol Eye Hospital for provision of specimens. Presented in part as a poster at the ARVO annual meeting, May 2011.

Author Contributions

Conceived and designed the experiments: CB KMM. Performed the experiments: CB EPD CSK SH TS NJT. Analyzed the data: CB EPD SJG. Contributed reagents/materials/analysis tools: KN JH TS NJT. Wrote the paper: CB KN JH KMM.

References

1. Meek KM (2009) Corneal collagen–its role in maintaining corneal shape and transparency. Biophysical Reviews 1: 83–93.
2. Connon CJ, Meek K (2003) Organization of corneal collagen fibrils during the healing of trephined wounds in rabbits. Wound Repair Regen 11: 71–78.
3. Hayes S, Young R, Boote C, Hawksworth N, Huang Y, et al. (2010) A structural investigation of corneal graft failure in suspected recurrent keratoconus. Eye 24: 728–734.
4. Kamma-Lorger CS, Hayes S, Boote C, Burghammer M, Boulton ME, et al. (2009) Effects on collagen orientation in the cornea after trephine injury. Molecular Vision 15: 378–385.
5. Cintron C, Schneider H, Kublin C (1973) Corneal scar formation. Exp Eye Res 17: 251–259.
6. Rawe IM, Meek KM, Leonard DW, Takahashi T, Cintron C (1994) Structure of corneal scar tissue: an X-ray diffraction study. Biophys J 67: 1743–1748.
7. Kamma-Lorger CS, Boote C, Hayes S, Albon J, Boulton ME, et al. (2009) Collagen ultrastructural changes during stromal wound healing in organ cultured bovine corneas. Experimental Eye Research 88: 953–959.
8. Dawson DG, Edelhauser HF, Grossniklaus HE (2005) Long-term histopathologic findings in human corneal wounds after refractive surgical procedures. Am J Ophthalmol 139: 168–178.
9. Melles GR, Binder PS (1990) A comparison of wound healing in sutured and unsutured corneal wounds. Arch Ophthalmol 108: 1460–1469.
10. Steele C (2009) Corneal wound healing: a review. Optometry Today 24: 28–32.
11. Farley MK, Pettit TH (1987) Traumatic wound dehiscence after penetrating keratoplasty. Am J Ophthalmol 104: 44–49.
12. Lang GK, Green WR, Maumenee AE (1986) Clinicopathologic studies of keratoplasty eyes obtained post mortem. Am J Ophthalmol 101: 28–40.
13. Morrison JC, Swan KC (1982) Full-thickness lamellar keratoplasty. A histologic study in human eyes. Ophthalmology 89: 715–719.
14. Morrison JC, Swan KC (1982) Bowman's layer in penetrating keratoplasties of the human eye. Arch Ophthalmol 100: 1835–1838.
15. Morrison JC, Swan KC (1983) Descemet's membrane in penetrating keratoplasties of the human eye. Arch Ophthalmol 101: 1927–1929.
16. Boote C, Hayes S, Abahussin M, Meek KM (2006) Mapping collagen organisation in the human cornea: left and right eyes are structurally distinct. Invest Ophthalmol Vis Sci 47: 901–908.
17. Meek KM, Quantock AJ (2001) The use of X-ray scattering techniques to determine corneal ultrastructure. Prog Retin Eye Res 20: 95–137.
18. Boote C, Dennis S, Newton RH, Puri H, Meek KM (2003) Collagen fibrils appear more closely packed in the prepupilliary cornea- optical and biomechanical implications. Investigative Ophthalmology and Visual Science 44: 2941–2948.
19. Meek K, Quantock AJ, Boote C, Liu CY, Kao WWY (2003) An X-ray scattering investigation of corneal structure in keratocan deficient mice. Matrix Biology 22: 467–475.
20. Boote C, Du Y, Morgan SR, Harris J, Kamma-Lorger CS, et al. (2012) Quantitative Assessment of Ultrastructure and Light Scatter in Mouse Corneal Debridement Wounds. Invest Ophthalmol Vis Sci 53: 2786–2795.
21. Meek KM, Boote C (2009) The use of x-ray scattering techniques to quantify the orientation and distribution of collagen in the corneal stroma. Prog Retin Eye Res 28: 369–392.
22. Borcherding MS, Blacik LJ, Sittig RA, Bizzel JW, Breen M, et al. (1975) Proteoglycans and Collagen Fibre Organisation in Human Corneoscleral Tissue. Experimental Eye Research 21: 59–70.
23. Boote C, Kamma-Lorger CS, Hayes S, Harris J, Burghammer M, et al. (2011) Quantification of collagen organization in the peripheral human cornea at micron-scale resolution. Biophys J 101: 33–42.

24. Aghamohammadzadeh H, Newton RH, Meek KM (2004) X-ray scattering used to map the preferred collagen orientation in the human cornea and limbus. Structure 12: 249–256.
25. Boote C, Dennis S, Huang Y, Meek K (2005) Lamellar orientation in human cornea in relation to mechanical properties. Journal of Structural Biology 149: 1–6.
26. Daxer A, Fratzl P (1997) Collagen fibril orientation in the human corneal stroma and its implications in keratoconus. Investigative Ophthalmology and Visual Science 38: 121–129.
27. Pramanik S, Musch DC, Sutphin JE, Farjo AA (2006) Extended long-term outcomes of penetrating keratoplasty for keratoconus. Ophthalmology 113: 1633–1638.
28. Calkins JL, Hochheimer BF, Stark WJ (1981) Corneal wound healing: holographic stress-test analysis. Invest Ophthalmol Vis Sci 21: 322–334.
29. Pettinelli DJ, Starr CE, Stark WJ (2005) Late traumatic corneal wound dehiscence after penetrating keratoplasty. Arch Ophthalmol 123: 853–856.
30. Vrana E, Builles N, Hindie M, Damour O, Aydinli A, et al. (2008) Contact guidance enhances the quality of a tissue engineered corneal stroma. J Biomed Mater Res A 84: 454–463.
31. Boyce BL, Grazier JM, Jones RE, Nguyen TD (2008) Full-field deformation of bovine cornea under constrained inflation conditions. Biomaterials 4: 4.
32. Farrell RA, McCally RL (2000) Corneal Transparency. In: Albert DM, Jakobiec FA, editors. Principles and Practice of Ophthalmology. Philadelphia: W.B. Saunders Company. 629–643.
33. Abahussin M, Hayes S, Knox Cartwright NE, Kamma-Lorger CS, Khan Y, et al. (2009) 3D collagen orientation study of the human cornea using X-ray diffraction and femtosecond laser technology. Invest Ophthalmol Vis Sci 50: 5159–5164.
34. Meek KM, Newton RH (1999) Organization of collagen fibrils in the corneal stroma in relation to mechanical properties and surgical practice. Journal of Refractive Surgery 15: 695–699.
35. Smelser GK, Polack FM, Ozanics V (1965) Persistence of donor collagen in corneal transplants. Exp Eye Res 4: 349–354.
36. Nguyen TD, Boyce BL (2011) An inverse finite element method for determining the anisotropic properties of the cornea. Biomech Model Mechanobiol 10: 323–337.
37. Sen DK, Mohan H, Gupta DK (1969) Bilateral peripheral sclero-cornea. Acta Ophthalmol (Copenh) 47: 1041–1043.
